# Supplementary material for: TPMM: three-component posterior mixture model enables robust inverton detection in low-depth metagenomes and suggests potential viral invertons
Source: Bioinformatics. 2026 Aug 21;42(Suppl 2):btag437. doi: 10.1093/bioinformatics/btag437 (PMC13501310; doi:10.1093/bioinformatics/btag437)
Supplement: Supplementary file 1 [file btag437_supplementary_data.pdf]

TPMM: Supplementary Materials

1. DEFINITION OF PAIRED-END (PE) AND SPANNING (SPAN)

To avoid ambiguity and to ensure consistency with PhaseFinder, we used the definitions of paired-end and spanning evidence described in PhaseFinder. The original description is reproduced below[1]:

"PhaseFinder uses two complementary strategies to detect invertible regions, paired-end and spanning. In the paired-end method, the goal is to find read pairs that overlap the junction of the inverting and non-inverting region that uniquely support either the F or R orientation. Reads that align equally well to both the F and R orientations are discarded as they are not informative for supporting either orientation. In the spanning method, the goal is to find reads that span the entire inverted repeat with at least 9 bp of overhang on either side. Therefore, spanning reads either support the F or R orientation. In rare cases where spanning reads could align to both the F and R orientations equally well, they are discarded."

To further clarify the distinction between these two approaches, we provide a schematic illustration in Figure S1.

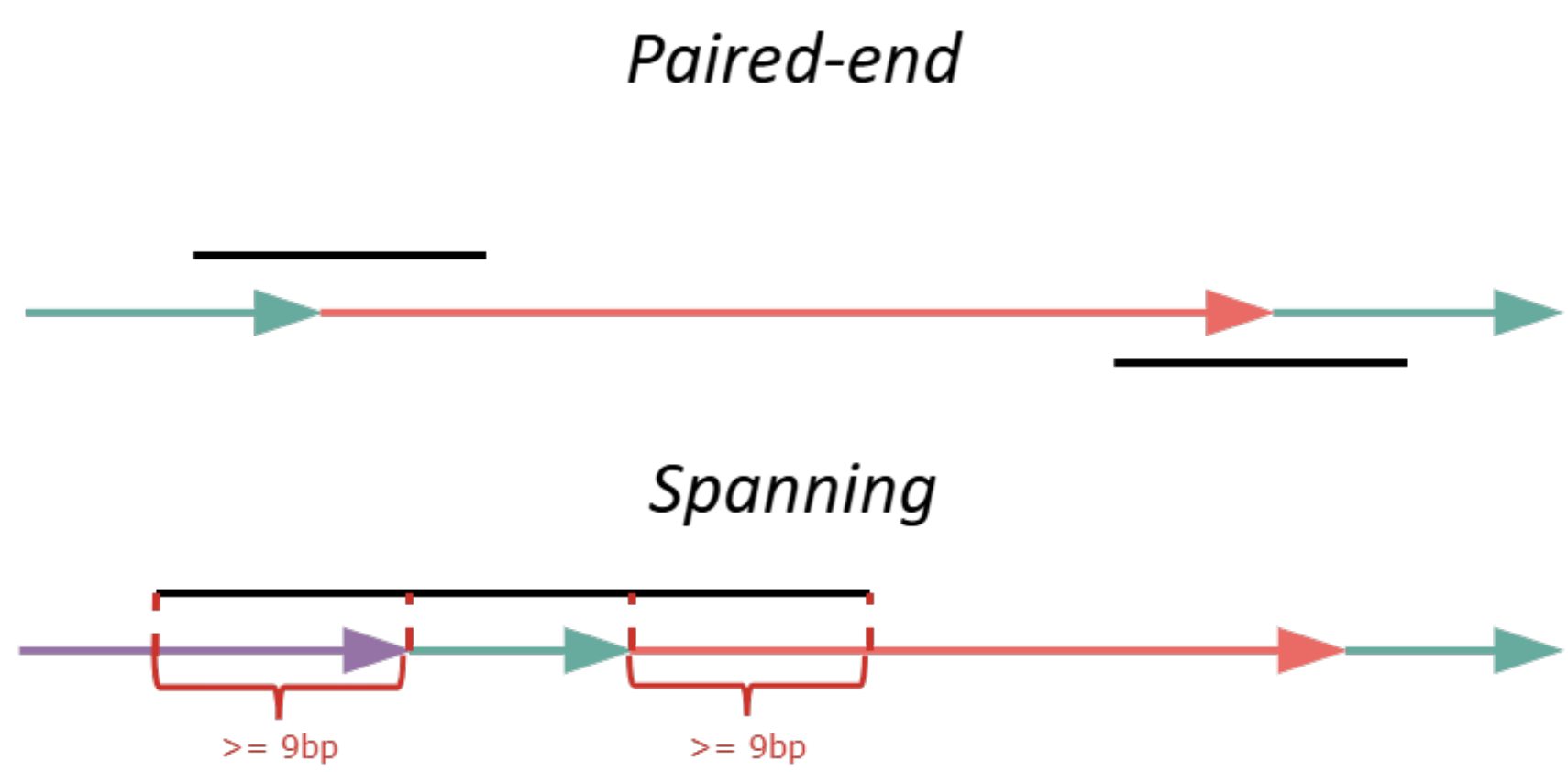

Fig. S1. Schematic diagram of Paired-end (Pe) and Spanning (Span)

2. MODEL SELECTION FOR MIXTURE COMPONENTS

To determine the optimal number of components in the binomial mixture model, we fitted models with two to five components and evaluated each using the Akaike Information Criterion (AIC) and Bayesian Information Criterion (BIC), defined as:

$$\text{AIC} = 2k - 2\ln(\hat{L}),$$

$$\text{BIC} = k\ln(n) - 2\ln(\hat{L}),$$

where k is the number of free parameters, $\hat{L}$ is the maximized likelihood evaluated on all scored observations, and n is the number of observations. Lower values indicate a better balance between goodness of fit and model complexity.

Table S1. Model selection criteria for mixture models with two to five components.

Components	Free parameters (k)	AIC	ΔAIC	BIC
2	5	336,461	–	336,503
3	8	263,474	72,987	263,540
4	11	245,361	18,113	245,452
5	14	233,680	11,681	233,796

The AIC and BIC measure the trade-off between model fit and complexity, with lower values indicating a better-fitting model; ΔAIC denotes the reduction in AIC relative to the previous model, with larger values indicating greater improvement in fit. The three component model was selected based on the elbow pattern observed in the AIC values, where the transition from two to three components yielded the largest improvement (ΔAIC = 72,987), while subsequent transitions contributed substantially less. The three-component model was further favored for its biological interpretability, with the three components corresponding naturally to a noise component, a low-probability inverton component, and a high-probability inverton component.

3. IDENTIFIABILITY CONSIDERATIONS FOR TPMM

TPMM contains latent signal components whose numerical labels are arbitrary unless an additional convention is imposed. In particular, for the two inversion signal components, exchanging the labels $k = 1$ and $k = 2$, together with their associated parameters, gives the same fitted distribution. This is the usual label-switching issue in latent mixture models and does not affect the likelihood or the fitted probabilities.

To assign consistent biological interpretations to the two signal components, we impose an ordering constraint on their average reverse-read probabilities across the two channels:

$$\frac{\theta_2^{\text{Pe}} + \theta_2^{\text{Span}}}{2} \geq \frac{\theta_1^{\text{Pe}} + \theta_1^{\text{Span}}}{2}. \tag{S1}$$

Under this convention, component $k = 1$ is defined as the low-probability inversion state and component $k = 2$ is defined as the high-probability inversion state. This constraint is used only to fix the component labels. It does not otherwise restrict the fitted distribution.

Beyond label switching, the interpretation of TPMM parameters requires standard non-degeneracy conditions. First, the two signal components must have distinct reverse-read probability profiles. For true invertons, where

$$p_1^c(i) = \theta_1^c, \qquad p_2^c(i) = \theta_2^c,$$

this requires that the parameter vectors

$$\boldsymbol{\theta}_1 = \left(\theta_1^{\text{Pe}}, \theta_1^{\text{Span}}\right), \qquad \boldsymbol{\theta}_2 = \left(\theta_2^{\text{Pe}}, \theta_2^{\text{Span}}\right)$$

are not identical. If $\boldsymbol{\theta}_1 = \boldsymbol{\theta}_2$, then the two signal components have the same emission distribution and cannot be distinguished from the observed data.

Second, each component must have nonzero representation in the data. If the mixing proportion or posterior support of one component is effectively zero, then the parameters associated with that component are weakly identified or not estimable from the data.

Third, sufficient read coverage is required in the relevant channels. When coverage is very low, or when all observations are concentrated at boundary values, the reverse-read probabilities can become weakly identified. Therefore, TPMM inference is interpreted under the assumption that the candidate region has enough informative paired-end and spanning-read observations.

Finally, when the ordering constraint holds with equality,

$$\frac{\theta_2^{\text{Pe}} + \theta_2^{\text{Span}}}{2} = \frac{\theta_1^{\text{Pe}} + \theta_1^{\text{Span}}}{2},$$

the low- and high-probability labels are not uniquely determined by the ordering criterion. In this degenerate case, the fitted distribution remains unchanged but the biological interpretation of the two labels becomes ambiguous. In practice, interpretable separation between the two inversion states requires a nonzero difference in their average reverse-read probabilities.

4. EM ESTIMATION AND POSTERIOR INFERENCE

The parameter set is

$$\Theta = \{\pi_0, \pi_1, \pi_2,\ \theta_1^{\mathrm{Pe}}, \theta_2^{\mathrm{Pe}}, \theta_1^{\mathrm{Span}}, \theta_2^{\mathrm{Span}},\ \beta_0, \beta_1\}.$$

We estimate Θ by expectation–maximization (EM), maximizing the summed log-likelihood

$$\mathcal{L}(\Theta) = \sum_{i=1}^{m} \ln P(\boldsymbol{x}_i \mid \Theta).$$

E-step. Given current parameters $\Theta^{(t)}$, responsibilities (posterior component probabilities) are

$$\begin{aligned}\gamma_i^{k\,(t)} &= P(z_i = k \mid \boldsymbol{x}_i, \Theta^{(t)}) \\ &= \frac{\pi_k^{(t)}\, P(\boldsymbol{x}_i \mid z_i = k, \Theta^{(t)})}{\sum_{j=0}^{2} \pi_j^{(t)}\, P(\boldsymbol{x}_i \mid z_i = j, \Theta^{(t)})}.\end{aligned}$$

We report $posterior\_noise = \gamma_i^0$, $posterior\_low = \gamma_i^1$, $posterior\_high = \gamma_i^2$, and

$$posterior\_non\_noise = 1 - \gamma_i^0.$$

M-step. Mixture weights are updated by

$$\pi_k^{(t+1)} \leftarrow \frac{1}{m} \sum_{i=1}^{m} \gamma_i^{k\,(t)} \qquad (k = 0, 1, 2).$$

For $k \in \{1, 2\}$, the channel-specific binomial parameters admit responsibility-weighted MLE updates:

$$\begin{aligned}p_k^{Pe,(t+1)} &\leftarrow \frac{\sum_{i=1}^{m} \gamma_i^{k\,(t)} R_i^{Pe}}{\sum_{i=1}^{m} \gamma_i^{k\,(t)} N_i^{Pe}}, \\ p_k^{Span,(t+1)} &\leftarrow \frac{\sum_{i=1}^{m} \gamma_i^{k\,(t)} R_i^{Span}}{\sum_{i=1}^{m} \gamma_i^{k\,(t)} N_i^{Span}}.\end{aligned}$$

The noise regression coefficients (β_0, β_1) are updated by maximizing the expected noise log-likelihood, which is equivalent to a weighted binomial logistic regression on aggregated totals:

$$R_i \mid N_i \sim \mathrm{Binomial}(N_i,\ \sigma(\beta_0 + \beta_1 x_i))\,, x_i = \ln(1 + N_i),$$

with observation weights $\gamma_i^{0\,(t)}$. We solve this weighted binomial-logit problem using iteratively reweighted least squares (IRLS) on the aggregated counts.

Table S2. EM algorithm for TPMM with two evidence channels and coverage-dependent noise.

Input: $\{R_i^{Pe}, N_i^{Pe}, R_i^{Span}, N_i^{Span}\}_{i=1}^{m}$; totals $R_i = R_i^{Pe} + R_i^{Span}$, $N_i = N_i^{Pe} + N_i^{Span}$; tolerance ϵ; max iterations T; number of restarts S.

Initialize: For each restart s: draw $\pi^{(0)} \sim \text{Dirichlet}(1,1,1)$; initialize $\theta_1^{\text{Pe}}, \theta_2^{\text{Pe}}$ and $\theta_1^{\text{Span}}, \theta_2^{\text{Span}}$ using empirical reverse-fraction quantiles plus small Gaussian perturbations; set β_0 from a low quantile of the total reverse fraction (default: 0.05) and $\beta_1 = 0$. Keep the restart with the largest final $\mathcal{L}$.

For $t = 0, 1, \ldots, T-1$ **do**

E-step: Compute $p_i^{noise} = \sigma(\beta_0^{(t)} + \beta_1^{(t)} \ln(1 + N_i))$. For each i, compute $\gamma_i^{k\,(t)}$ from the mixture likelihood using both channels.

M-step: Update $\pi_k^{(t+1)} \leftarrow m^{-1} \sum_i \gamma_i^{k\,(t)}$.

Update $p_k^{Pe,(t+1)}$ and $p_k^{Span,(t+1)}$ for $k \in \{1,2\}$ by responsibility-weighted MLEs.

Update $(\beta_0^{(t+1)}, \beta_1^{(t+1)})$ by weighted binomial-logit IRLS on (R_i, N_i, x_i) with weights $\gamma_i^{0\,(t)}$.

Enforce label stability: if $\frac{\theta_2^{\text{Pe}} + \theta_2^{\text{Span}}}{2} < \frac{\theta_1^{\text{Pe}} + \theta_1^{\text{Span}}}{2}$, swap labels $1 \leftrightarrow 2$ (including π_1, π_2).

Convergence: Stop if $|\mathcal{L}^{(t+1)} - \mathcal{L}^{(t)}| < \epsilon$.

End for

Output: $\widehat{\Theta}$ and posteriors $\gamma_i^k = P(z_i = k \mid \boldsymbol{x}_i, \widehat{\Theta})$.

A. Fitting strategy

Coverage varies substantially across different candidates. To stabilize fitting, we estimate Θ on a high-coverage subset selected by quantiles of N_i (default: $q_{low} = 0.90$ to $q_{high} = 0.99$) after filtering with total coverage $N_i \geq N_{\min}$ (default: $N_{\min} = 8$). We then compute posteriors for all candidates passing the minimum coverage threshold using the fitted $\widehat{\Theta}$.

B. Implementation details

We implement the EM algorithm in Python using numerically stable log-space computations (log-sum-exp) for responsibilities. Probabilities are clipped away from 0 and 1 (default clipping to $[10^{-10}, 1 - 10^{-10}]$) to avoid undefined logarithms. EM uses multiple random restarts (default: 25) and returns the solution with the highest final log-likelihood; termination uses an absolute log-likelihood tolerance (default: 10^{-6}) and a maximum iteration cap (default: 500). The weighted noise logistic regression is fitted by IRLS on aggregated binomial counts (R_i, N_i) with covariate $\ln(1 + N_i)$ and weights γ_i^0, consistent with maximizing the expected noise-component log-likelihood.

C. Sensitivity of TPMM Parameter Estimation to the Number of Candidate Loci

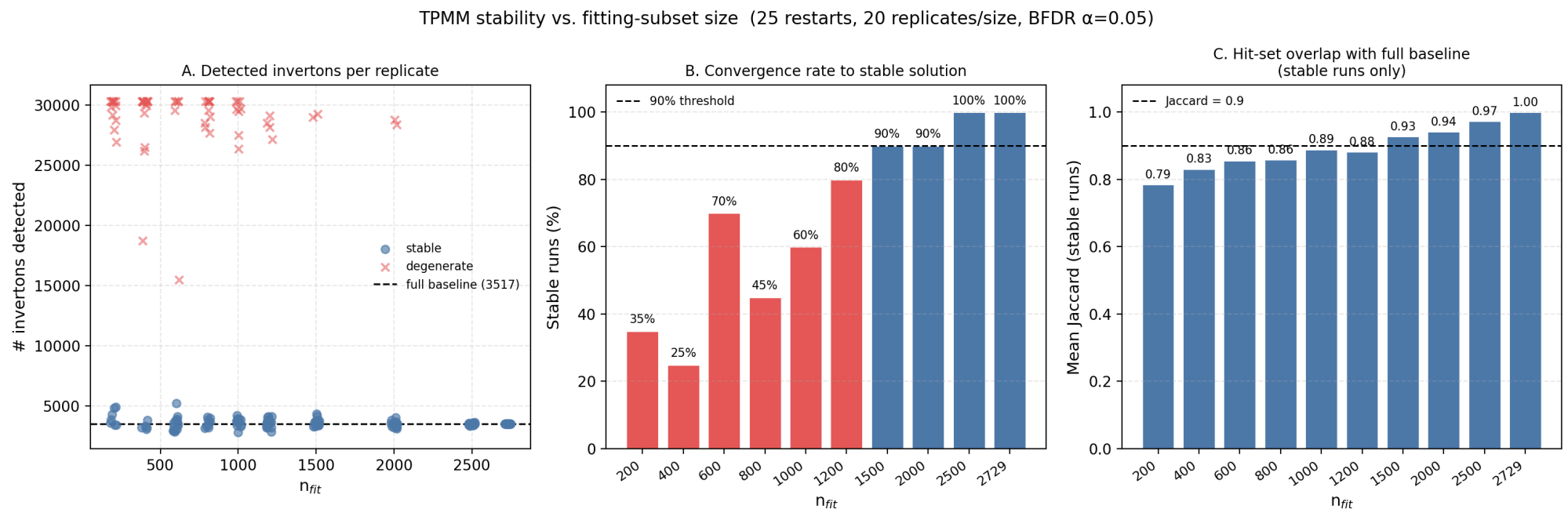

Fig. S2. Sensitivity of TPMM to the number of candidate loci used for EM parameter estimation using human dataset. (A) Detected invertons per replicate. Blue circles: stable solutions; red crosses: degenerate solutions in which the noise component collapsed. Dashed line: full result (3,517 invertons). (B) Fraction of stable runs at each loci count. $\geq 90\%$ stability (dashed line) is first reached at 1,500 candidate loci. (C) Among stable runs, mean Jaccard index with the full result. Inverton identity is largely invariant to the number of candidate loci once convergence is achieved (Jaccard ≥ 0.93 at $\geq 1{,}500$ loci).

5. DETAILED DEFINITION OF DOWNSTREAM GENES

We denote the start and end coordinates of an identified inverton as a and b, respectively. For a CDS predicted by Prodigal, let c and d represent the start and end coordinates, with the strand denoted as "-1" (minus) or "1" (plus). The downstream genes of an inverton are defined as follows:

$$0 < c - b \leq \omega, strand = 1$$
$$or$$
$$0 < a - d \leq \omega, strand = -1$$

6. TOOLS USED FOR DATA PREPROCESSING

- Trimmomatic (v0.39) [2]
- Bowtie2 (v2.3.5.1) [3–5]
- MEGAHIT (v1.2.9) [6]

Subset	Count
LEADING	20
TRAILING	20
SLIDINGWINDOW	4:20
AVGQUAL	20
HEADCROP	1
MINLEN	100

Table S3. Parameters of Trimmomatic

7. CONSISTENCY BETWEEN POSTERIOR AND CUMULATIVE BFDR

We further examined the concordance between posterior-based ranking and cumulative BFDR. To this end, we plot two quantities as scatter points in Figure S3:

- **Red scatter points:** the relationship between P_{true} and $-log_{10}(BFDR_{cum})$

- **Blue scatter points:** the relationship between candidate rank(sorting by P_{true} in descending order) and
  $-log_{10}(BFDR_{cum})$,

where each point represents one candidate. As shown in Figure S3, both scatter points lie on smooth trajectories. Specifially, The red points indicate that P_{true} increases rapidly with $-\log_{10}(BFDR_{cum})$ and then saturates in the high-evidence area., while the ranks progress systematically toward the top of the list. Importantly, this concordance is structurally expected: $BFDR_{cum}$ is computed as a prefix average of the ranked local false discovery rate(lfdr), and $P_{true} = 1 - lfdr$ is directly complementary to lfdr. This concordance supports using P_{true} as a continuous credibility score for ranking candidates, and shows its compatibility with BFDR-based thresholding. Consequently, one can obtain interpretable probabilistic outputs while performing principled selection at a target BFDR level. In particular, for downstream analysis, we defined high-confidence positives using the thresholds $BFDR_{cum} < 0.05$, which is marked in Figure S3.

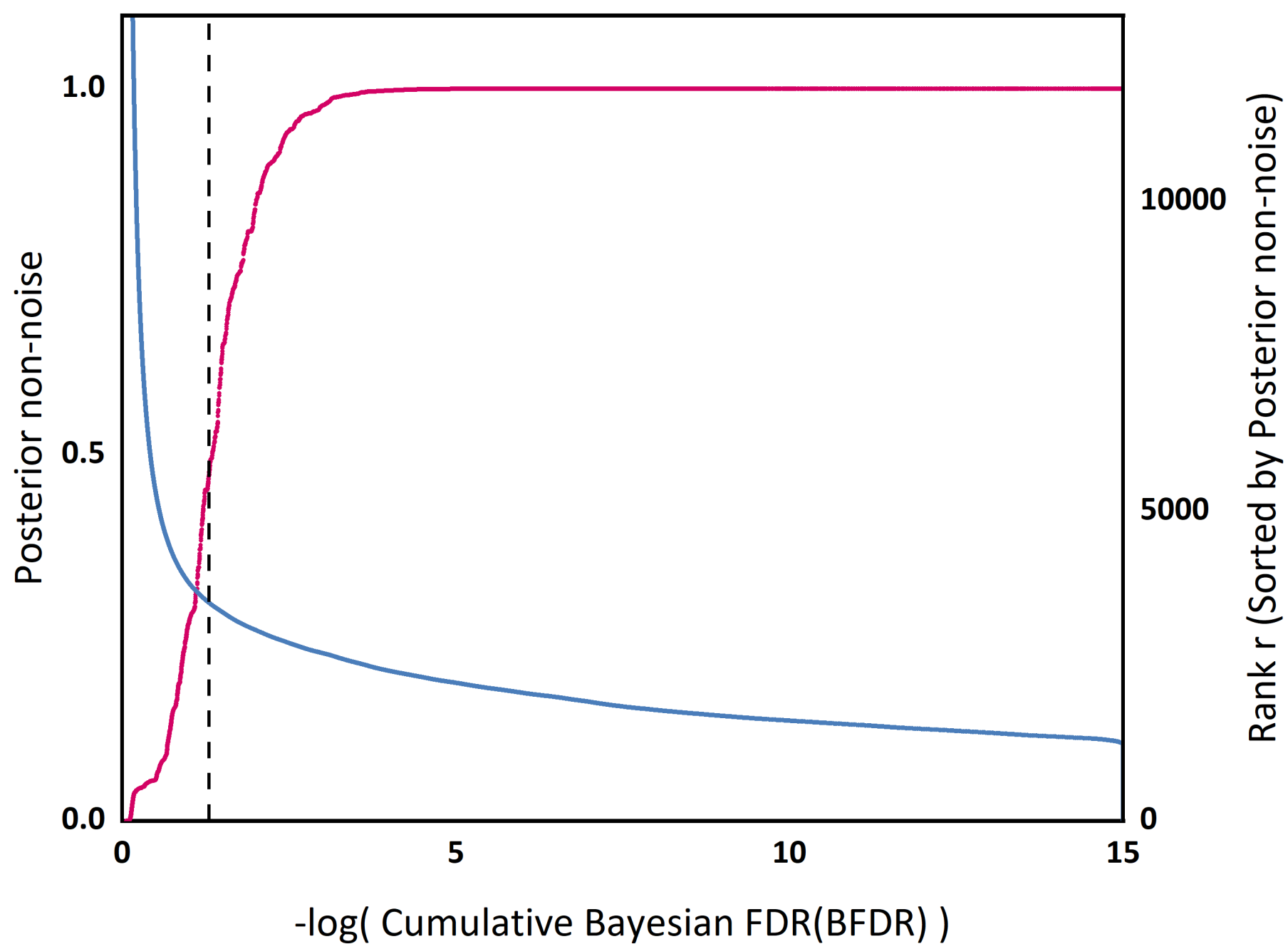

Fig. S3. Posterior–BFDR ranking curve of human dataset

8. COMPARISON OF THE IDENTIFIED NUMBERS BETWEEN TPMM AND PHASEFINDER

Figure S4 shows a Venn diagram of the resulting call sets, where the blue circle represents TPMM positives and the red circle represents PhaseFinder positives. Overall, the two methods exhibit high concordance: most detected invertons are shared. In the human gut dataset, 2719 invertons are detected by both methods, whereas 395 are unique to PhaseFinder and 797 are unique to TPMM; thus, the combined method-specific calls are approximately one third of the overlap. A similar pattern is observed in the rat gut dataset.

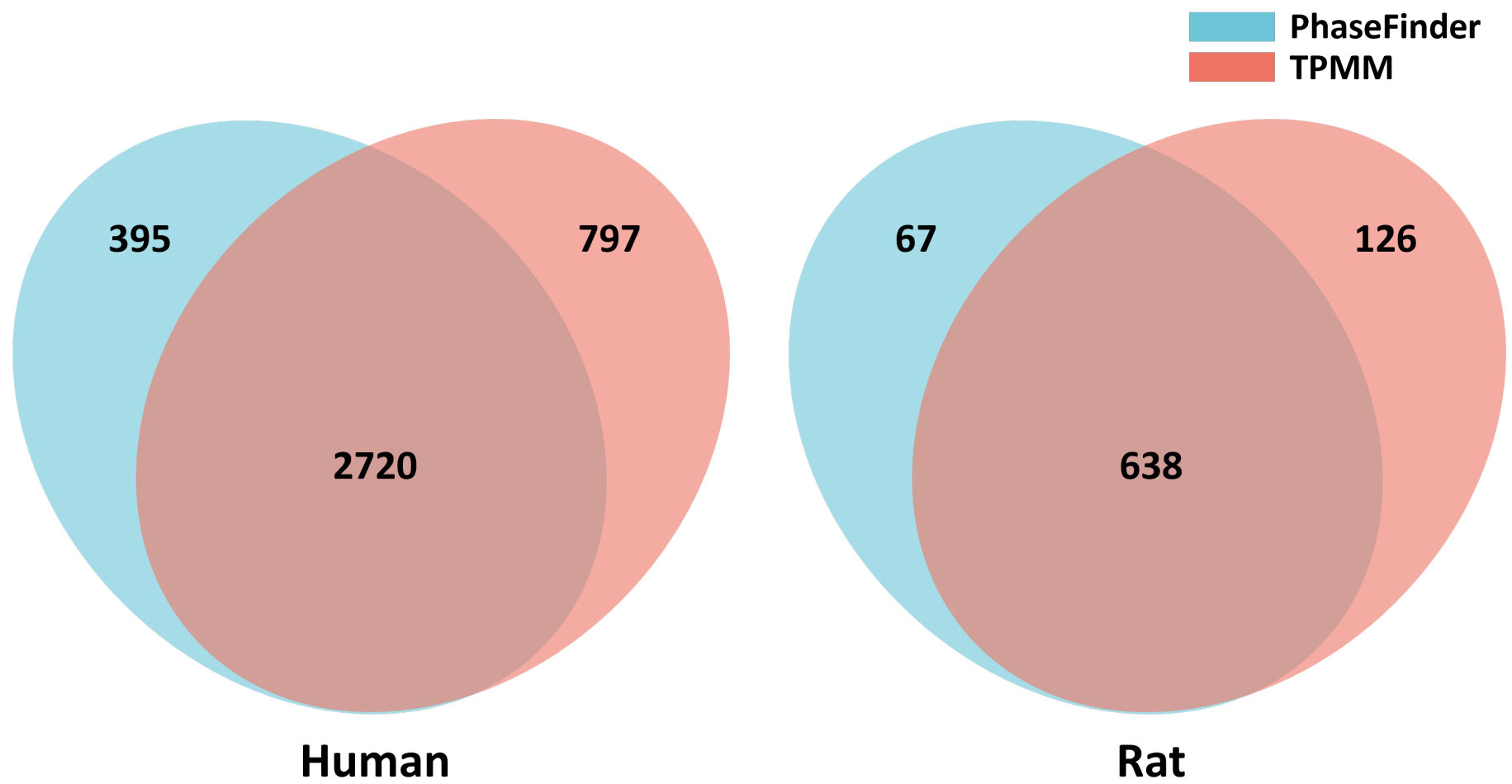

Fig. S4. Venn diagram comparison of positive inverton sets in human and rat datasets detected by TPMM and PhaseFinder

9. FALSE POSITIVE ANALYSIS UNDER DOWNSAMPLING

Since experimentally validated ground truth is not available for the human gut metagenomic dataset, we used I_{ref} as an operational proxy for ground truth:

$$I_{\text{ref}} = I^{\text{Full}}_{\text{TPMM}} \cap I^{\text{Full}}_{\text{PhaseFinder}}.$$

Calls overlapping I_{ref} were considered reference-supported; calls outside I_{ref} were counted as putative false positives. We defined I_{total} as the total number of candidates after pre-filtering ($R_i^{\text{Pe}} + R_i^{\text{Span}} \geq 1$), I_{detected} as the number of method calls, and $I_{\text{supported}}$ as calls overlapping I_{ref}. Proxy Recall and proxy FPR were computed as:

$$\text{proxy Recall} = \frac{I_{\text{supported}}}{|I_{\text{ref}}|}, \qquad \text{proxy FPR} = \frac{I_{\text{detected}} - I_{\text{supported}}}{I_{\text{total}}}.$$

This analysis is conservative: I_{ref} is stringent but incomplete, and calls outside it may represent genuine invertons missed by PhaseFinder due to hard read-count thresholds or coverage differences, rather than true false positives.

Results for the human gut dataset ($I_{\text{total}} = 37{,}519$) are summarized in Supplementary Table S4. TPMM achieved higher recall than PhaseFinder across all downsampling conditions while maintaining comparable proxy FPR. To further characterize TPMM calls outside I_{ref}, we examined their posterior probabilities (Supplementary Table S5). The mean posterior noise probability γ^0 remained low across all conditions (0.121, 0.133, and 0.151 at DS20, DS40, and DS80, respectively), confirming that these calls represent high-confidence inverton signals rather than noise artifacts. This is further consistent with the read-evidence pattern in Figure 2 of the main text, where TPMM-unique calls concentrate in bins with high R_i/N_i but low absolute N_i, the regime in which PhaseFinder's hard thresholds cause missed detections.

To assess robustness under severe downsampling, the DS20 experiment was repeated five times with different random seeds (Supplementary Table S6). TPMM consistently maintained higher recall with comparable proxy FPR across all replicates. Across all six DS20 runs, 1,086 putative invertons were detected in at least three runs and 835 in at least four, confirming the stability and consistency of these results.

Table S4. Reference-based false positive analysis on the human gut dataset under downsampling.

Downsampling	Method	I_{detected}	$I_{\text{supported}}$	proxy Recall (%)	proxy FPR (%)
DS20	TPMM	1093	1015	37.3	0.21
DS20	PhaseFinder	797	764	28.1	0.09
DS40	TPMM	2055	1748	64.3	0.82
DS40	PhaseFinder	1498	1388	51.0	0.30
DS80	TPMM	3045	2458	90.4	1.56
DS80	PhaseFinder	2678	2355	86.6	0.86

Table S5. Posterior probability summary of TPMM calls outside I_{ref} under downsampling.

Condition	n	γ^0 (noise)		γ^1 (low)		γ^2 (high)	
		mean	median	mean	median	mean	median
DS20	78	0.121	0.074	0.201	0.002	0.678	0.837
DS40	307	0.133	0.109	0.210	0.003	0.656	0.765
DS80	587	0.151	0.134	0.311	0.009	0.537	0.732

TPMM calls not overlapping I_{ref} at each downsampling level. γ^0, γ^1, γ^2: posterior probabilities for the noise, low-probability, and high-probability inverton components, respectively.

Table S6. Reference-based false positive analysis of five repeated DS20 downsampling experiments of TPMM on the human gut dataset.

Replicate	I_{detected}	$I_{\text{supported}}$	proxy Recall (%)	proxy FPR (%)
Replicate 1	1074	1018	37.4	0.15
Replicate 2	1118	1055	38.8	0.17
Replicate 3	1276	1177	43.3	0.26
Replicate 4	1208	1123	41.3	0.23
Replicate 5	1083	1033	38.0	0.13

For each repeated DS20 downsampling experiment, detected invertons were compared with I_{ref}.

10. PUTATIVE VIRAL INVERTONS FROM ANOTHER HUMAN GUT DATASET

To seek additional evidence for the presence and functional potential of viral invertons, we analyzed another human gut metagenomic dataset. We selected the week 0 metagenomic sequencing data from HMP2 IBD samples[7]. Applying the identical viral contig filtering pipeline described in the main text, we successfully identified two putative invertons located on high-confidence viral contigs. Both of them locate entirely within the boundaries of CDSs.

While one of the two identified invertons failed to show significant alignment to any known viral sequences via BLASTN, investigation of another inverton revealed a biologically meaningful

phenomenon. Using BLASTN for nucleotide sequence annotation and BLASTP for protein functional prediction, we found that this viral contig aligns closely with a *Caudoviricetes* sp. reference genome (Accession: BK020372). In its F orientation, the CDS containing the inverton aligned to two distinct tail measure proteins, showing a stronger preference for accession ID DAJ59527.1 (Max score: 1774) over accession ID DAI79821.1 (Max score: 1724).

To evaluate the functional impact of this structural variation, we performed an *in silico* inversion of the invertible segment and re-annotated the modified sequence. Strikingly, in this inverted orientation, the relative alignment hierarchy of the two tail measure proteins was reversed. The modified sequence now displayed a higher preference for DAI79821.1 (Max score: 1933) compared to DAJ59527.1 (Max score: 1931). This dynamic shift in the primary protein match suggests that the inversion event may generate alternative structural variants of the tail measure protein, potentially expanding the functional repertoire or altering the infection dynamics of the virus.

REFERENCES

1. X. Jiang, A. B. Hall, T. D. Arthur, *et al.*, "Invertible promoters mediate bacterial phase variation, antibiotic resistance, and host adaptation in the gut," Science **363**, 181–187 (2019).
2. A. M. Bolger, M. Lohse, and B. Usadel, "Trimmomatic: a flexible trimmer for illumina sequence data," Bioinformatics **30**, 2114–2120 (2014).
3. B. Langmead, C. Wilks, V. Antonescu, and R. Charles, "Scaling read aligners to hundreds of threads on general-purpose processors," Bioinformatics **35**, 421–432 (2018).
4. B. Langmead, C. Trapnell, M. Pop, and S. L. Salzberg, "Ultrafast and memory-efficient alignment of short dna sequences to the human genome," Genome Biol. **10**, R25 (2009).
5. B. Langmead and S. L. Salzberg, "Fast gapped-read alignment with bowtie 2," Nat. Methods **9**, 357–359 (2012).
6. D. Li, C.-M. Liu, R. Luo, *et al.*, "Megahit: an ultra-fast single-node solution for large and complex metagenomics assembly via succinct de bruijn graph," Bioinformatics **31**, 1674–1676 (2015).
7. L. M. Proctor, H. H. Creasy, J. M. Fettweis, *et al.*, "The integrative human microbiome project," Nature **569**, 641–648 (2019).
